# Triggering Receptor Expressed on Myeloid Cell 2 R47H Exacerbates Immune Response in Alzheimer’s Disease Brain

**DOI:** 10.3389/fimmu.2020.559342

**Published:** 2020-09-25

**Authors:** Olena Korvatska, Kostantin Kiianitsa, Alexander Ratushny, Mark Matsushita, Neal Beeman, Wei-Ming Chien, Jun-Ichi Satoh, Michael O. Dorschner, C. Dirk Keene, Theo K. Bammler, Thomas D. Bird, Wendy H. Raskind

**Affiliations:** ^1^Department of Psychiatry and Behavioral Sciences, University of Washington, Seattle, WA, United States; ^2^Department of Immunology, University of Washington, Seattle, WA, United States; ^3^Seattle Biomedical Research Institute and Institute for Systems Biology, Seattle, WA, United States; ^4^Department of Medicine, Division of Medical Genetics, University of Washington, Seattle, WA, United States; ^5^Department of Bioinformatics and Molecular Neuropathology, Meiji Pharmaceutical University, Tokyo, Japan; ^6^Department of Pathology, University of Washington, Seattle, WA, United States; ^7^Department of Environmental and Occupational Health Sciences, Seattle, WA, United States; ^8^Department of Neurology, University of Washington, Seattle, WA, United States; ^9^Geriatric Research, Education and Clinical Center, Veteran Affairs Puget Sound Health Care System, Seattle, WA, United States; ^10^Mental Illness Research, Education and Clinical Center, Department of Veteran Affairs, Seattle, WA, United States

**Keywords:** microglia, neurodegeneration, aging, inflammation, interferon type I response, NKG2D ligands, senescence

## Abstract

The R47H variant in the microglial triggering receptor expressed on myeloid cell 2 (TREM2) receptor is a strong risk factor for Alzheimer’s disease (AD). To characterize processes affected by R47H, we performed an integrative network analysis of genes expressed in brains of AD patients with R47H, sporadic AD without the variant, and patients with polycystic lipomembranous osteodysplasia with sclerosing leukoencephalopathy (PLOSL), systemic disease with early-onset dementia caused by loss-of-function mutations in TREM2 or its adaptor TYRO protein tyrosine kinase-binding protein (TYROBP). Although sporadic AD had few perturbed microglial and immune genes, TREM2 R47H AD demonstrated upregulation of interferon type I response and pro-inflammatory cytokines accompanied by induction of NKG2D stress ligands. In contrast, PLOSL had distinct sets of highly perturbed immune and microglial genes that included inflammatory mediators, immune signaling, cell adhesion, and phagocytosis. TREM2 knockout (KO) in THP1, a human myeloid cell line that constitutively expresses the TREM2- TYROBP receptor, inhibited response to the viral RNA mimetic poly(I:C) and phagocytosis of amyloid-beta oligomers; overexpression of ectopic TREM2 restored these functions. Compared with wild-type protein, R47H TREM2 had a higher stimulatory effect on the interferon type I response signature. Our findings point to a role of the TREM2 receptor in the control of the interferon type I response in myeloid cells and provide insight regarding the contribution of R47H TREM2 to AD pathology.

## Introduction

A proactive role of brain immune cells, microglia, in the pathogenesis of Alzheimer’s disease (AD) gained recognition after the discovery of AD-associated variants in the triggering receptor expressed on myeloid cell 2 (*TREM2*) gene that is only expressed on immune cells of myeloid origin. Further support came from a large-scale genome-wide association study that identified multiple additional immune risk factors (odds ratio 0.9–1.15) (1). Many of these immune molecules have either microglia-specific expression (e.g., CD33, the MS4As, INPP5D, and SPI1) or are enriched in microglia (e.g., ABCA7, CR1, and CLU). Of all microglial genetic AD risk factors, the TREM2 R47H variant confers the highest risk of disease development, with a risk similar to that of APOEε4 (odds ratio 4.5–2.6) (2, 3). Loss-of-function mutations in the TREM2 receptor or its adaptor, TYRO protein tyrosine kinase-binding protein (TYROBP), cause polycystic lipomembranous osteodysplasia with sclerosing leukoencephalopathy (PLOSL; Online Mendelian Inheritance in Man # 221770), a recessive systemic disorder that presents with early-onset dementia (4) or familial frontotemporal dementia (5).

TREM2 is involved in multiple aspects of myeloid cell function and interaction with other cell types (6). In microglia, it regulates cell chemotaxis (7), enhances phagocytosis (8, 9), and influences microglia survival, proliferation, and differentiation (10). The complexity of TREM2 functioning is revealed in cell/tissue content-dependent responses to stimuli of different natures, as well as their timing. For instance, whereas isolated TREM2-deficient cells increased production of pro-inflammatory molecules *in vitro* (8, 11), studies of intact tissues demonstrated that both pro- and anti-inflammatory roles of TREM2 in brain immunity are dependent on timing after stimulation (12), stage of pathology (13), and genetic background (14). In addition, a soluble form of TREM2, a product of cleavage or alternative splicing, may have a separate function as an extracellular signaling molecule that promotes cell survival (15, 16). TREM2 binds lipids and lipoproteins, including the known AD risk factors ApoE and ApoJ/CLU (17–19), and mediates myelin clearance (20). Also, TREM2 binds amyloid beta-peptide (Aβ) activating microglia cytokine production and degradation of an internalized peptide (9).

By exome sequencing of families affected with late-onset AD, we identified multiple carriers of the TREM2 R47H variant (21). TREM2 R47H AD patients demonstrated a shortened course of the disease, pronounced synucleinopathy, changed microglial morphology, and decreased level of the microglial marker Iba1, suggesting that compromised TREM2 signaling has a strong effect on microglia function in the disease. Herein, we performed RNA expression profiling of brain tissues from subjects with TREM2 R47H AD, sporadic AD without the variant (sAD), and normal age-matched controls to assess the effect of R47H variant on brain immune gene networks. We also analyzed brain tissues of PLOSL patients with bi-allelic loss-of-function mutations in TREM2 or TYROBP. To further investigate the role of TREM2 and its R47H variant, we knocked out the gene in the THP1 cells, a human myeloid line that expresses a functional TREM2-TYROBP receptor.

## Materials and Methods

### Subjects

#### Familial Alzheimer’s Disease and Frontotemporal Dementia Patients With R47H

Through the University of Washington (UW) AD Research Center, we acquired brain autopsy material of nine TREM2 R47H carriers from four unrelated families affected with AD or frontotemporal dementia (Supplementary Tables 1, 2). Flash-frozen brain tissues from two R47H carriers were used for RNA-seq analyses. RNA isolated from formalin-fixed, paraffin-embedded (FFPE) sections of eight R47H carriers with AD were used for gene expression by NanoString nCounter.

#### Sporadic Alzheimer’s Disease and Controls

Frozen and fixed autopsy tissues from neuropathologically confirmed AD cases and aged non-demented controls were obtained from the UW Neuropathology Core Brain Bank (Supplementary Tables 1, 2).

#### PLOSL Cases Caused by Loss-Of-Function Mutations in TREM2-TYROBP Receptor

Brain tissues were obtained from the UW Neuropathology Core Brain Bank and the Meiji Pharmaceutical University, Tokyo, Japan. One PLOSL patient was homozygous for a missense variant D134G in *TREM2* (4), another patient was homozygous for a splice site mutation, c.482 + 2T > C, in *TREM2* (22), and two were homozygous for c.141delG in *TYROBP* (23) (Supplementary Tables 1, 2). FFPE sections were used for RNA isolation and NanoString nCounter analysis.

Under protocols approved by the Institutional Review Boards of the UW and the Meiji Pharmaceutical University, all subjects had previously given informed consent to share and study autopsy material. All methods for processing and analyzing the brain autopsy tissues followed relevant guidelines and regulations.

### Cell Culture and Cytokine Stimulation

A human microglia cell line HMC3 and human myeloid cell lines THP1, U937, and MOLM13 were obtained from the American Type Culture Collection. Cells were cultured in Dulbecco’s modified Eagle medium (HMC3) or RPMI medium (the others) supplemented with 10% fetal bovine serum. Pro-inflammatory stimulation with lipopolysaccharide (LPS; Sigma, #055:B5, 0.5 μg/ml) and interferon-gamma (IFNγ; PeproTech, #300-02, 150 U/ml) was performed for 24 h. Stimulations with interleukin (IL)-4 (PeproTech, #200-04, 20 ng/ml) or IL-10 (PeproTech, #200-10, 20 ng/ml) were similarly performed for 24 h.

### Genome Editing of TREM2 With CRISPR/CAS9

A THP1 derivative that stably expresses Cas9 (THP1-CAS9) was generated using a lentiviral construct (Addgene, lentiCas9-Blast, #52962) after blasticidin selection. Single guide RNAs (sgRNAs) for targeting *TREM2* were selected using the online CRISPR design tool (crispr.mit.edu) (24). THP1-CAS9 cells were nucleofected with sgRNAs using a protocol recommended for THP1 (Lonza, Amaxa 4D Nucleofector, Protocol #292). Cutting efficiency for each guide was measured using GeneArt Genomic Cleavage Detection Kit (ThermoFisher, #A24372). Based on % indel efficiency, sgRNA within exon 2 (sense: ACTGGTAGAGACCCGCATCA) was chosen for subsequent experiments. TREM2-negative cells were enriched by cell sorting after the immunostaining of live cells. Colonies grown from single sorted cells were sequenced; clones with frame-shifting indel mutations inactivating all *TREM2* alleles were tested for the absence of protein by Western blotting.

### TREM2 Cloning and Overexpression

Coding sequences of human *TREM2* (NM_018965.3), common variant (CV), and R47H variant, were synthesized and cloned into a doxycycline-inducible lentiviral pCW57-MCS1-2A-MCS2 vector (Addgene, #71782). THP1 TREM2 knockout (KO) cells were transduced with lentiviral particles expressing either of wild-type TREM2, R47H TREM2, or green fluorescent protein (GFP) proteins (Addgene; pCW57-GFP-2A-MCS, #71783). After 2 weeks of puromycin selection, resistant cells were induced with doxycycline (100 ng/ml) and tested for TREM2 and GFP expression by real-time quantitative PCR (qRT-PCR) and fluorescence-activated cell sorting analyses.

### Phagocytosis Assay

*In vitro* aggregation of an Aβ (Aβ_1__–__42_, Bachem #H-1368, 100 μM in phosphate-buffered saline) was carried out by incubation for 4 days at 37°C. The insoluble pellet was precipitated and labeled by pHrodo Red according to the manufacturer’s protocol (Life Technologies, #P36600). THP1 monocytes were plated at 5 × 10^5^/ml density; TREM2 expression was induced by 100 ng/ml doxycycline overnight. Cells were incubated with 0.25 μg/ml Aβ-pHrodo for 3 h at 37°C and collected and washed in ice-cold phosphate-buffered saline with 1% bovine serum albumin. Uptake of Aβ-pHrodo was measured on the LSRII Flow cytometer (BD Biosciences) and data analyzed by FlowJo. Phagocytosis was measured as the mean fluorescence intensity of the internalized pHrodo.

### THP1 Differentiation Into Macrophages and Stimulation of Interferon Type I Response

THP1 differentiation to macrophages was performed using 5 ng/ml phorbol 12-myristate 13-acetate (PMA) for 48 h followed by 24 h of recovery culturing without PMA (25, 26). IFN I response was induced by high molecular weight poly(I:C) complexes with LyoVec transfection reagent (Invivogen, tlrl-piclv, 500 ng/ml) or with a combination of poly(I:C)/LyoVec and IFNβ (PeproTech, #300-02, 100 Units/ml) for 24 h. When indicated, TREM2 or GFP expression was induced by 100 ng/ml doxycycline 16–18 h before the IFN I response stimulation.

### Real-Time Quantitative PCR Analysis

Total RNA from harvested cells was isolated with RNAeasy kit (Qiagen; #74106); complementary DNA was synthesized with SensiFast (Bioline, BIO-65053). TaqMan^®^ Fast Advanced Master Mix and the following TaqMan Gene Expression Assays (ThermoFisher, #4331182) were used for qRT-PCR: MICB: Hs00792952_m1, IFNB: Hs01077958_s1, IRF7: Hs01014809_g1, IFIH1: Hs00223420_m1, and reference gene TBP: Hs00427620_m1. Assays were performed on a StepOnePlus real-time PCR machine and analyzed with StepOne software (Applied Biosystems) using the 2[−Delta Delta C(T)] method (27). All measurements were performed in technical duplicates.

### Antibodies and Immunostaining

Live cells were immunostained with fluorescent polyclonal antibodies to TREM2 (R&D Systems #MAB17291) and analyzed by fluorescence-activated cell sorting. For Western blotting, 50 μg of whole-cell lysates was resolved on a polyacrylamide gel, transferred to polyvinylidene fluoride membrane, and stained with polyclonal TREM2 antibodies (Cell Signaling Technology, #91068) at 1:1,000 dilution.

### Gene Expression Analyses

#### Whole Transcriptome RNA-seq of Brain Tissues

Total RNA was isolated from flash-frozen brain tissues, and ribosomal RNA was depleted using the Ribo Zero Gold Magnetic system (Epicenter/Illumina, San Diego, CA, United States). RNA-seq libraries were prepared with the ScriptSeq v2 kit (Epicenter) and subjected to paired-end sequencing (2 × 100 bp) on an Illumina HiSeq 2500 instrument. A mean read number of 6 × 10^7^ was generated per sample. The following data analysis tools were used: FastQC 0.9.6 for visualizing read QC https://www.bioinformatics.babraham.ac.uk/projects/fastqc; Bowtie 2.2.1 https://sourceforge. net/projects/bowtie-bio/files/bowtie2/2.2.1/and TopHat 2.0.11 https://ccb.jhu.edu/software/tophat/index.shtml for read alignment and splice site discovery; Cufflinks 2.1.1 http://cole- trapnell-lab.github.io/cufflinks/releases/v2.1.1/for transcript assembly and quantification; MISO 0.5.2 https://sbgrid.org/software/titles/miso for quantitating isoform expression levels; and cummeRbund 2.0.0 http://compbio.mit.edu/cummeRbund and IGV 2.3.14 http://software.broadinstitute.org/software/igv/download for data visualization.

#### Gene Expression Analysis by nCounter System (NanoString Technologies)

Total RNA was isolated from FFPE samples. For each sample, four 10 μm thick FFPE sections were processed using the Recover All RNA isolation kit (Ambion, Termo Fisher) following the manufacturer’s protocol. RNA yield and fragment length distribution were measured by Qubit Fluorimeter (Molecular Probes, Termo Fisher) and 2100 Bioanalyzer (Agilent Technologies). The typical yield was 0.8–1 μg per 10 μm section. As expected, RNAs from archived FFPE samples were highly degraded (RNA integrity number 2–3); however, more than 80% of RNA species were over 80 bp allowing analysis by NanoString technology (hybridization with the 70 nucleotides gene-specific probe). Total RNA (1 μg) was used for hybridization on a NanoString nCounter platform according to the manufacturer’s instruction. Samples in which >50% genes had raw counts below the cutoff of 20 (average of eight negative controls plus two standard deviations, 95% confidence interval) were excluded from the analysis.

For gene expression analysis, we used the PanCancer Immune panel, which contains 730 immunity-related and cancer genes together with 40 validated housekeeping genes and a custom CodeSet containing 30 genes designed at NanoString Technologies. All probe sets were produced in a single batch. Data were normalized and log2 transformed using the nSolver 3.0 Analysis Software (NanoString Technologies). In brief, normalization was performed in two steps: (1) technical normalization using positive control spikes on each panel and (2) input amount normalization using the mean expression of 30 housekeeping genes selected by a geNorm algorithm on the basis of stability of their expression through all experimental groups. All transcripts with raw counts below the threshold 20 [average of eight negative controls plus two SD (∼95% confidence interval)] were labeled as undetected. Only genes detected at a level of 30 or more row counts in at least 50% of our samples were included in the analysis (*N* = 493).

### Gene Set and Pathway Analyses

Gene set analysis (GSA) (28) was used to calculate significant enrichment of known transcriptional signatures in gene expression data from RNA-seq. We used 10,295 gene sets from the Molecular Signature Database version 4.0 (29). False discovery rate (FDR) and *P-*value estimates were computed for each gene set based on 1,000 separate permutation distributions.

To identify biologically relevant pathways in differentially expressed (DE) genes from NanoString nCounter data, we used Ingenuity Pathway Analysis (Ingenuity Systems, Qiagen) and Metascape^1^. Ingenuity Pathway Analysis queries a proprietary knowledge database and calculates the significance of genes/pathways enrichment using Fisher’s exact test. Metascape queries public databases [Reactome, MSigDB, Gene Ontology (GO), Kyoto Encyclopedia of Genes and Genomes Pathway (KEGG), and CORUM], using the hypergeometric test to calculate enrichment. Additionally, Metascape performs an analysis of protein–protein interactions (data from the BioGrid database) in which subnetworks with high local network connectivity are found using the molecular complex detection algorithm (30). Eight hundred genes on the NanoString panel were used as a baseline for enrichment calculations. Visualization of protein–protein association networks that include known protein interaction and a functional connection was done using the STRING database^2^ (31).

### Statistical Analysis

Hypergeometric distributions were evaluated using the online calculator https://systems.crump.ucla.edu/hypergeometric/index.php. Other statistical tests were performed using GraphPad Prism software.

## Results

### NanoString Profiling Reveals Perturbed Immune, Microglial and AD-Related Genes in TREM2 R47H AD and Few Alterations in sAD

Hippocampi of TREM2 R47H AD patients (*N* = 8) were compared with those of sAD (*N* = 15) and normal aged controls (*N* = 17); the latter two groups were chosen to minimize covariation with age, sex, disease stage, postmortem interval, and RNA quality (Supplementary Tables 1, 2). Hippocampus is one of the most affected brain regions in AD, and in a previous study, we observed the most pronounced changes in microglial markers in the hippocampi of R47H carriers (21). We used NanoString nCounter, a mid-throughput nucleic acid hybridization-based platform suitable for analysis of RNA from FFPE material. A predesigned PanCancer Immune panel (770 genes) contained the main human immunity-related pathways; of those, 156 genes were microglia-specific, i.e., expressed at least 10-fold higher in microglia than in other brain cell populations (32). Of 800 genes on the panel, 551 had detectable expression in FFPE samples, and 493 of these, including 134 microglia-specific genes (MGs), were included in the analysis.

At present, the NanoString nCounter is the method of choice for profiling RNA from FFPE-archived tissues. A recent study of FFPE AD brains found no major effect of postmortem interval and RNA degradation on gene expression data (33). Also, this and other studies reported high concordance of NanoString expression data in frozen and FFPE material from the same case (34), and we confirmed this for our samples and experimental setting. RNA isolated from flash-frozen or FFPE tissue of the same individual showed high Pearson’s correlation of gene expression (*r*^2^ = 0.84). Additionally, the robustness of the assay was tested in the “discovery mode” using RNA from FFPE hippocampi of patients with X-linked parkinsonism with spasticity (Online Mendelian Inheritance in Man # 300911; *N* = 2), a monogenic disorder caused by a variant in the *ATP6AP2* gene (21). The mutation alters ATP6AP2 splicing, resulting in the abnormally high expression of the isoform that skips exon 4 (e3-e5). We found that e3-e5 was significantly increased in X-linked parkinsonism with spasticity brains, as previously shown on RNA prepared from fresh blood cells of carriers (Supplementary Figure 1). Thus, even in a small group of FFPE samples, NanoString nCounter reliably detects gene expression changes caused by a mutation of predicted effect.

**FIGURE 1:**
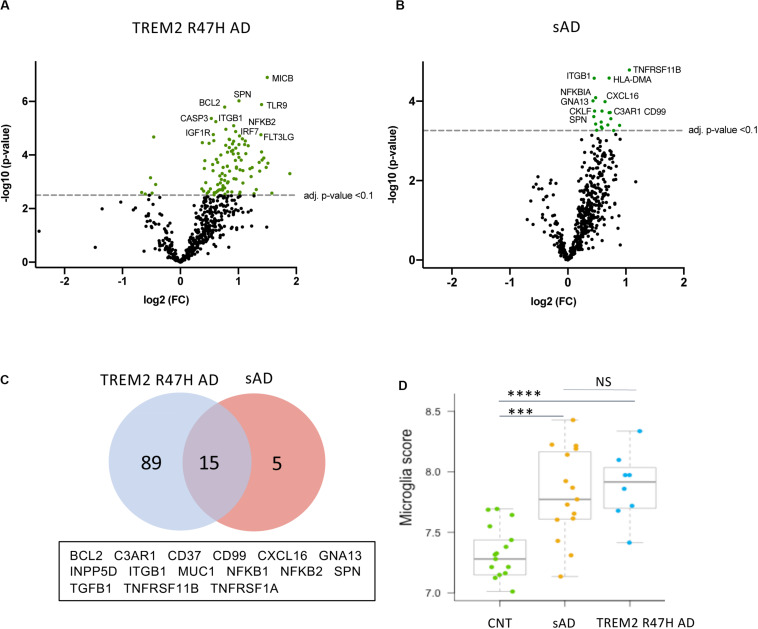
Differentially expressed (DE) genes in hippocampi of TREM2 R47H AD and sAD patients. **(A–D)** Volcano plots displaying DE genes in TREM2 R47H AD **(A)** and sAD **(B)** against a baseline of gene expression in controls. *y*-Axis corresponds to the log_10_(*p*-value), and the *x*-axis displays the log_2_ (FC, fold changes) value. **(C)** Venn diagram and list of shared DE genes (FDR < 0.1) between TREM2 R47H AD and sAD. **(D)** Microglia cell scores were calculated by nSolver (v.3) as an average log-transformed expression of 134 microglia-specific genes that allow comparison of cell abundance across samples (71). Each unit increase in a cell score calculated from log2 transformed data corresponds to a doubling of microglia abundance (NS, non-significant; ^∗∗∗^*p*-value < 0.001, ^****^p-value < 0.0001, one-way ANOVA, and Tukey’s multiple comparison test).

The TREM2 R47H AD group had the largest number of DE genes (104 vs. 20 in sAD, FDR < 0.1, Figures 1A,B). 75 percent of DE genes in sAD were shared with TREM2 R47H AD (Figure 1C and Supplementary Table 3). We measured the relative abundance of microglial cells in the brain tissue by scoring the expression of 134 MGs (MG signature, Figure 1D). Both AD groups showed a similar increase in microglia cellularity compared with controls, suggesting that the observed increase in immune genes in TREM2 R47 AD is not due to an increased number of microglia.

**FIGURE 2:**
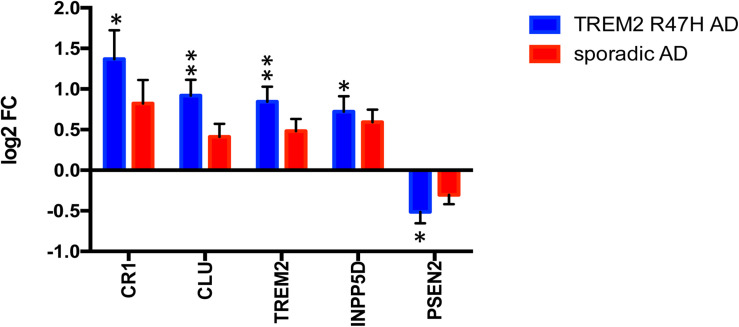
Perturbed expression of AD genes/risk factors in TREM2 R47H AD. AD-associated genes altered (FDR < 0.1) in at least one condition against a baseline of gene expression in controls are shown. Data are presented as mean ± SD (^∗^adj *p*-value < 0.05; ^∗∗^adj *p*-value < 0.01); *y*-axis displays log2 (FC, fold changes) value.

We also assessed whether TREM2 R47H affects transcript levels of TREM2 itself, as well as other known AD genes and risk factors. Five of 16 genes with measurable expression were perturbed in TREM2 R47H AD, whereas none reached significance in sAD (Figure 2). Four genes, including TREM2, were upregulated, and PSEN2 was downregulated. Thus, the R47H variant has a profound effect on the expression of AD risk factors.

### Pro-inflammatory Immune Networks and Pathways Are Activated in TREM2 R47H AD Brains

**FIGURE 3:**
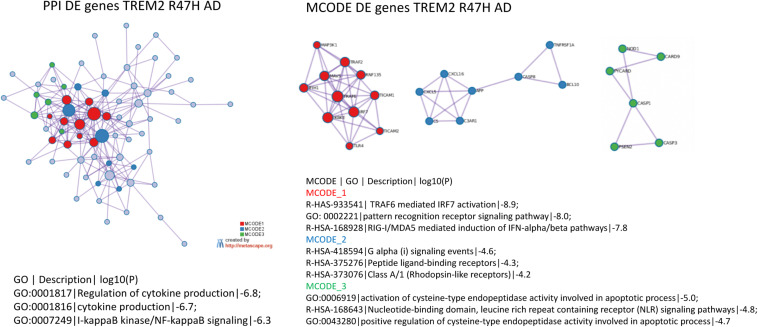
Protein–protein interaction networks and its tightly connected cores (molecular complex detection) formed by differentially expressed genes in TREM2 R47H AD. Protein–protein interaction, molecular complex detection, and Gene Ontology (GO) term enrichment were determined using Metascape (www.metascape.org). 800 genes on NanoString panel were used as a background for enrichment calculations. Tables with the corresponding GO term enrichment are shown. Column heading in table: GO, GO ID; Description: Representative GO term; Log10(P), *p*-value for enrichment.

The most perturbed pathways in TREM2 R47H AD patients comprised “WNT/Ca^++^ Signaling,” “Role of RIG1-like Receptors in Antiviral Innate Immunity,” “Inflammasome Pathway,” and “Neuroinflammation Signaling Pathway” (Table 1). Analysis of the protein–protein interaction network revealed enriched GO terms corresponding to the regulation of cytokine production and I-kappaB kinase/NF-kappaB signaling and identified three functional modules corresponding to type I interferon response, ligand-receptor interaction, and a module formed by PSEN2 and mediators of apoptosis (Figure 3). In contrast, sAD produced a heterogeneous group of overrepresented pathways driven by the tumor necrosis factor (TNF) receptor superfamily members (TNFRSF11b and TNFRSF1A) and the NF-κB complex and its regulators (Table 1). These findings corroborated a meta-analysis of sAD transcriptomes that identified the TNF receptor superfamily genes, such as TNFRSF11B and TNFRSF1A, and components of NFKB signaling, such as NFKBIA, NFKB1, and RELA, among the top, upregulated pathways in sAD (35).

**TABLE 1:** Top canonical pathways from Ingenuity pathway analysis of DE genes in TREM2 R47H AD and sAD.

**Ingenuity canonical pathways**	**-log(*p*-value)**	**zScore**	**Ratio**	**Genes**
**TREM2 R47H AD**				
Wnt/Ca + pathway	3.17	2.83	0.67	NFKB2, NFATC3, NFATC4, NFKB1, NFATC2, EP300, CREBBP, NFATC1
Role of RIG1-like receptors in antiviral innate immunity	2.73	3.00	0.50	MAVS, NFKB2, TRAF2, NFKB1, IRF7, IFIH1, EP300, CREBBP, CASP8, TRAF6, IKBKE
Inflammasome pathway	2.64	2.65	0.64	TLR4, NFKB2, NFKB1, NOD2, CASP1, CASP8, PYCARD
Neuroinflammation signaling pathway	2.45	3.54	0.31	TGFB1, NFATC2, TNFRSF1A, TICAM1, IL6R, TLR9, TREM2, CSF1R, TLR4, IRF7, CREBBP, TRAF6, NFATC4, CCL3, NFKB1, JAK2, EP300, CASP1, HLA-DOB, APP, MFGE8, NFATC1, PYCARD, NFKB2, NFATC3, PSEN2, BCL2, TICAM2, CASP3, CD200, CASP8, IKBKE
**sAD**				
Induction of apoptosis by HIV1	3.74	0.82	0.25	NFKB2, TNFRSF11B, NFKB1, TNFRSF1A, BCL2, NFKBIA
OX40 Signaling pathway	3.25	1.00	0.21	NFKB2, NFKB1, HLA-DMA, HLA-DRB3, BCL2, NFKBIA
Ceramide signaling	3.14	1.34	0.25	NFKB2, TNFRSF11B, NFKB1, TNFRSF1A, BCL2
PI3K/AKT signaling	3.08	1.64	0.19	NFKB2, NFKB1, INPP5D, ITGB1, BCL2, NFKBIA

### TREM2 R47H AD Brain Manifests Activation of Antiviral Response Genes and “Induced-Self” NKG2D Ligands

**FIGURE 4:**
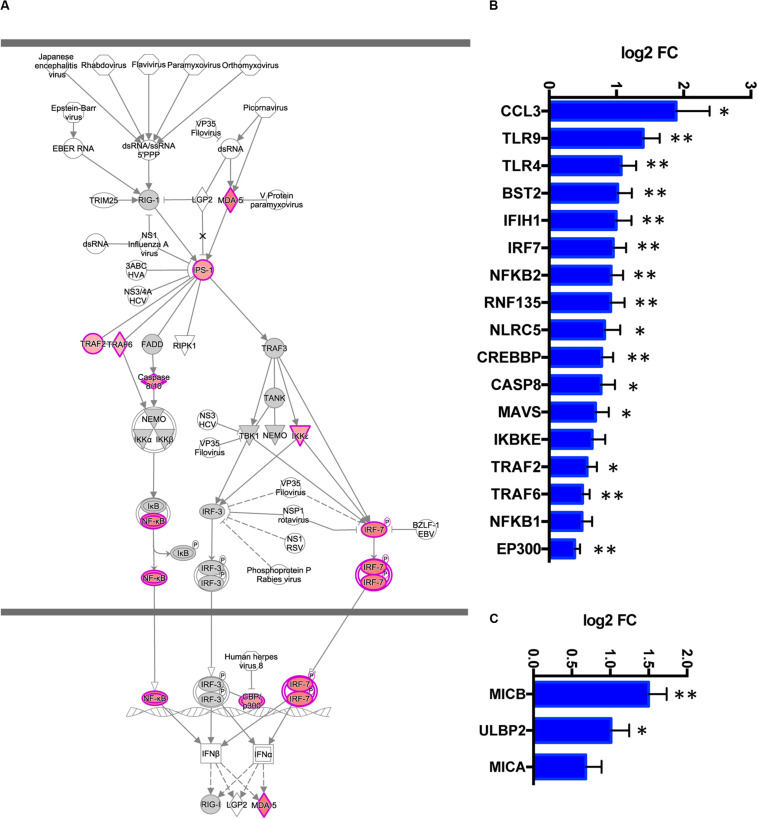
TREM2 R47H AD features upregulated interferon type I response and MHC class I-like NKG2D ligands. **(A)** Role of RIG1-like receptors in antiviral innate immunity, Ingenuity Pathway Analysis-generated diagram shows perturbed gene expression in TREM2 R47H AD brains. Red denotes upregulated genes; gray, not changed compared to control brains; white, not assessed. Perturbed expression (FDR < 0.1) of IFN I type response/antiviral genes **(B)** and MHC class I-like NKG2D ligands. **(C)** Data are presented as mean ± SD (^∗^adj *p*-value < 0.05; ^∗∗^adj *p*-value < 0.01); *y*-axis displays log2 (FC, fold changes) value.

The activated antiviral IFN I response and upregulated pro-inflammatory cytokines were the top immune signatures in TREM2 R47H brains (Table 1 and Figure 4). We also identified additional upregulated genes known to act in the antiviral response (RNF135/Riplet, TLR9, TLR4, and BST2/tetherin) that were not part of the canonical IFN I response signature (Figure 4B). During IFN I response, multiple downstream IFN-stimulated genes (ISGs) are upregulated. We, therefore, queried a database of ISG (INTERFEROME) (36) and found that DE genes in TREM2 R47H AD were ISG-enriched (hypergeometric test *p*-value is 0.03).

Dysregulated IFN I response activates multiple pro-inflammatory molecules causing cellular stress and cytotoxicity (37, 38). In TREM2 R47H AD brains, we observed upregulation of several MHC class I-like NKG2D ligands (MICB, MICA, and ULBP2, Figure 4C). These molecules are known as “induced self” because their expression on the cell surface is induced by diverse stress agents, such as viruses, heat shock, or oxidative damage. In the brain, MICB is expressed primarily by microglia (32), and it is the most significantly upregulated gene in TREM2 R47H AD (Figure 1A). We tested whether MICB may be upregulated in response to pro-inflammatory stimulation using a panel of human cell lines that share a myeloid origin with microglia. Stimulation with a combination of LPS and IFNγ upregulated MICB expression in all tested cell lines, whereas stimulation with type 2 cytokines IL-4 or IL-10 had no effect (Supplementary Figure 2).

### TREM2 R47H-Associated Immune Signatures Are Upregulated in Brain Transcriptomes of Carriers

We also had flash-frozen unfixed hippocampus tissue suitable for RNA-Seq analysis from two of the TREM2 R47H carriers. To assess the top affected pathways at the transcriptome-wide level, we sequenced the total RNA of these two TREM2 R47H carriers and three normal age-matched controls. Table 2 shows the top 1% of differentially regulated gene sets in the brains of TREM2 R47H carriers (23 of 2,076 sets identified by GSA) (28). Of the 16 most upregulated sets, 10 belonged to immune process/function, such as cytokine metabolism, antiviral response/TRAF6-mediated IRF7 activation, and immune response. The second most upregulated group consisted of four signatures related to cell cycle/mitosis. Seven most downregulated sets were related to ion and amino acid transport, endocytosis, glutamine metabolism, and synaptic transmission (Table 2).

**TABLE 2:** Top gene sets in TREM2 R47H brains are enriched by immunity-related GO terms.

**Gene set**	**GO term**	**GSA RNA-seq this study**	**GSA Blalock et al. 2004 (39)**		
			
		**TREM2 R47H**	**AD severe**	**AD moderate**	**AD incipient**
		
		**GSA score**			
**Upregulated**					
MODULE_424	Regulation of immune response (GO:0050776)	1.41	0.16	0.08	–0.03
Spindle and kinetochore	Mitotic nuclear division (GO:0007067)	1.28	–0.37	–0.49	–0.69
BIOCARTA_CYTOKINE_PATHWAY	Regulation of cytokine production (GO:0001817); positive regulation of JAK-STAT cascade (GO:0046427)	1.18	–0.05	0.18	–0.15
CYTOKINE_BIOSYNTHETIC_PROCESS	Regulation of cytokine biosynthetic process (GO:0042035)	1.15	0.01	0.08	0.00
CYTOKINE_METABOLIC_PROCESS	Regulation of cytokine biosynthetic process (GO:0042035)	1.15	0.01	0.08	0.00
SU_THYMUS	Immune system process (GO:0002376)	1.13	0.19	–0.21	–0.06
FINETTI_BREAST_CANCER_KINOME_RED	Mitotic cell cycle process (GO:1903047)	1.13	–0.04	–0.20	–0.54
REACTOME_TRAF6_MEDIATED_IRF7_ACTIVATION	Innate immune response (GO:0045087)	1.09	0.66	0.03	0.12
REGULATION_OF_CYTOKINE_SECRETION	Regulation of cytokine secretion (GO:0050707)	1.09	0.10	0.05	–0.01
REGULATION_OF_CYTOKINE_BIOSYNTHETIC_PROCESS	Regulation of cytokine biosynthetic process (GO:0042035)	1.08	–0.04	0.06	–0.06
YAMASHITA_LIVER_CANCER_WITH_EPCAM_DN	Steroid metabolic process (GO:0008202)	1.08	–0.06	0.00	–0.03
MODULE_478	Regulation of immune response (GO:0050776)	1.03	–0.12	–0.08	–0.26
CROMER_TUMORIGENESIS_UP	Extracellular matrix organization (GO:0030198)	1.03	0.01	–0.05	–0.06
MODULE_315	Mitotic cell cycle process (GO:1903047)	1.02	–0.57	–0.46	–0.60
KARAKAS_TGFB1_SIGNALING	Spindle checkpoint (GO:0031577)	1.01	0.09	–0.02	–0.10
CHEN_ETV5_TARGETS_SERTOLI	Immune system process (GO:0002376)	1.00	0.14	–0.21	0.13
**Downregulated**					
L_AMINO_ACID_TRANSMEMBRANE_TRANSPORTER_ACTIVITY	Amino acid transport (GO:0006865)	–1.35	–0.75	–0.21	–0.20
Oxidative phosphorylation (COX and ATPases)	Hydrogen ion transmembrane transport (GO:1902600)	–1.19	–1.70	–1.57	–0.60
MODULE_307	Hydrogen ion transmembrane transport (GO:1902600)	–1.19	–1.72	–1.49	–0.59
BIOCARTA_NDKDYNAMIN_PATHWAY	Endocytosis (GO:0006897), synaptic transmission (GO:0007268)	–1.05	–1.05	–0.79	–0.23
BIOCARTA_CACAM_PATHWAY	Protein phosphorylation (GO:0006468); synaptic transmission (GO:0007268)	–1.05	–0.83	–0.88	–0.60
LEIN_LOCALIZED_TO_DISTAL_AND_PROXIMAL_ DENDRITES	Activation of phospholipase C activity (GO:0007202); synaptic transmission (GO:0007268)	–1.04	–0.72	–0.49	–0.25
GLUTAMINE_FAMILY_AMINO_ACID_METABOLIC_PROCESS	Glutamine family amino acid metabolic process (GO:0009064)	–1.01	–0.37	–0.28	–0.16

*Shown are the top 1% of sets up-or downregulated in carriers of TREM2 R47H, GSA scores ≥ 1 or ≤ -1 (GSA of RNA-seq data, this study). Enrichment of the same gene sets in sporadic AD was assessed separately by performing GSA of the publicly available dataset of the three sAD cohorts (GSA Blalock et al., 2004) (39).*

To distinguish whether the upregulation of immune genes was a characteristic of AD pathology (effect of the disease) or due to abnormal function of the TREM2 immune receptor (effect of mutation), we performed GSA analysis on sAD data from the Blalock study [GDS810 (39)]. This dataset has been used in several meta-analyses of differential gene expression in AD (40–43). The study compared hippocampi of the non-demented control group (*N* = 9) with three progressive stages of AD: “incipient” (*N* = 7), “moderate” (*N* = 8), and “severe” (*N* = 7). The TREM2 R47H and sAD groups shared several downregulated sets (Table 2), and their enrichment scores increased with disease progression. Notably, immune sets upregulated in the TREM2 R47H group were not enriched in sAD.

### Bi-Allelic Loss-of-Function Mutations in TREM2 or TYROBP Cause Gross Perturbation of Microglial Networks in Brains of PLOSL Patients

**FIGURE 5:**
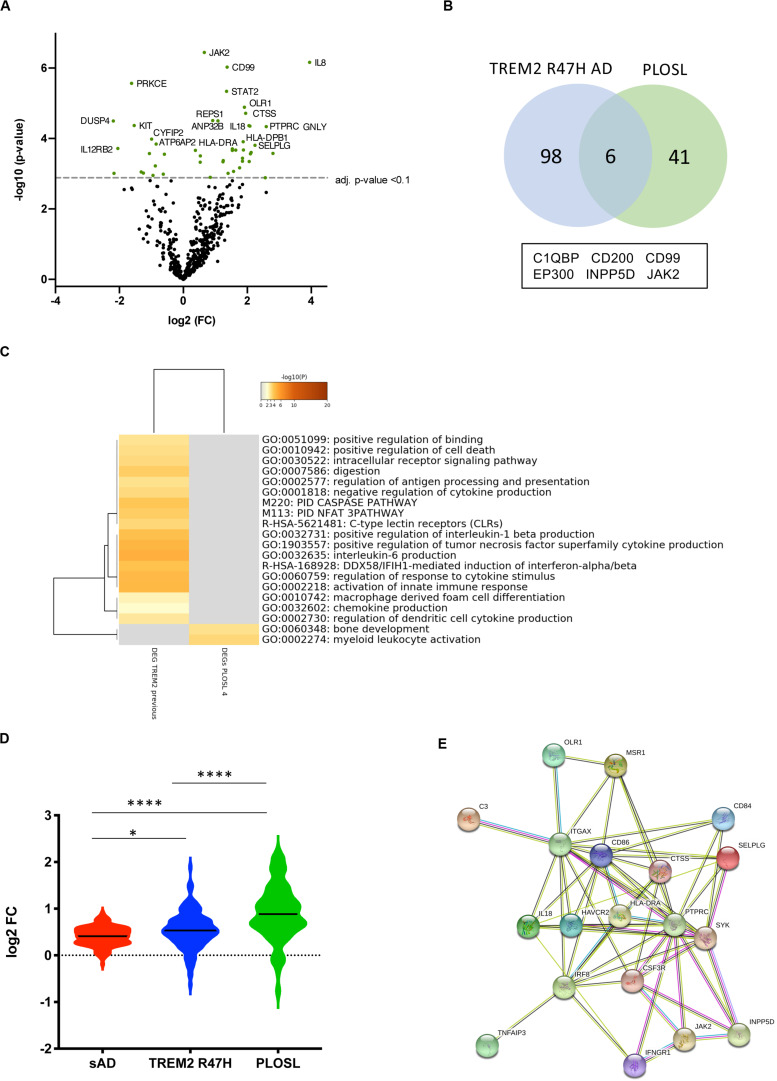
Differentially expressed (DE) and microglia-specific genes in PLOSL. **(A)** Volcano plots displaying DE genes in PLOSL against a baseline of gene expression in controls. **(B)** Venn diagram and list of shared DE genes (FDR < 0.1) between TREM2 R47H AD and PLOSL. **(C)** Enriched ontology clusters in DE TREM2 R47H AD and PLOSL. Metascape cluster analysis of GO term enrichment among DE genes (FDR < 0.1) between TREM2 R47H AD and PLOSL. Eight hundred genes on the NanoString panel were used as a background for enrichment calculations. GO ID, Description: Representative GO term; Log10(P) – *p*-value for enrichment. **(D)** Microglia-specific genes (MG) expression is highly perturbed in PLOSL as compared with AD groups. Distribution of fold changes in MG (*N* = 134); *X*-axis displays log2 of fold changes between gene expression in cases vs. controls (^∗^*p*-value < 0.05; ^****^p-value < 0.0001, one-way ANOVA, Tukey’s multiple comparison test); **(E)** Protein–protein association network formed by differentially expressed MG in PLOSL, as visualized by STRING database (http:/string-db.org).

PLOSL patients with bi-allelic mutations inactivating either subunit of the TREM2-TYROBP receptor develop an identical disease phenotype (4). This prompted us to compare changes in microglial and immune gene networks caused by the receptor complex loss with the effect of the TREM2 R47H variant. We analyzed samples from four PLOSL patients with loss-of-function mutations in TREM2 (*N* = 2) (4, 22) or TYROBP (*N* = 2) (23) by NanoString nCounter. DE genes in PLOSL (Figure 5A and Supplementary Table 4) were enriched for MGs (40.4 vs. 27% of expected distribution, hyper-geometric test, *p* = 0.027) consistent with the microglial origin of this disorder. In contrast, DE genes in TREM2 R47H AD were not MG-enriched (30 vs. 27% of expected distribution, hyper-geometric test, *p* = 0.21), suggesting that multiple cell types have contributed to the activation of an immune response in this group. PLOSL and TREM2 R47H AD shared only six DE genes, less than nine expected by chance, and no common pathways (Figures 5B,C). Top enriched pathways in PLOSL were “myeloid cell activation involved in immune response,” “bone development,” and “Fc-gamma Receptor-mediated Phagocytosis.” We also compared fold changes of expression of all MG expressed in PLOSL and AD brains. PLOSL showed the highest magnitude of MG perturbation, followed by TREM2 R47H AD (Figure 5D). All 19 DE MGs in PLOSL formed a tight network orchestrated by the IRF8 transcription factor. The network was enriched with pro-inflammatory (HLA-DRA, IL18, TNFAIP3, and ITGAX) and immune signaling molecules (SYK, JAK2, INPP5D, and PTPRC), endo-phagocytosis mediators (C3, MSR1), and cell adhesion/motility receptors (CSF3R, ITGAX, OLR1, CD84, and SELPLG, Figure 5E).

### TREM2 R47H Restores Defective Phagocytosis of Aggregated Amyloid Beta-Peptide in THP1 TREM2 Knockout Cells

**FIGURE 6:**
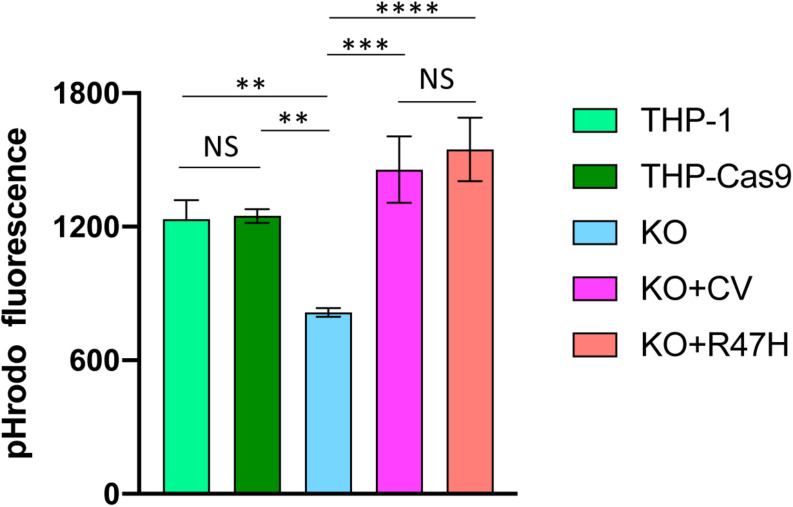
Phagocytosis of aggregated pHrodo-labeled amyloid beta-peptide. THP-1, unmodified cells; THP-Cas9, THP1 stably expressing Cas9 nuclease; KO, TREM2 knockout; KO + CV, KO + R47H, KO expressing common variant (CV) or R47H TREM2, respectively. All cells were treated with doxycycline, then incubated with pHrodo-labeled Aβ. Data are shown as mean of biological triplicates ± SD (NS, non-significant; ^∗∗^*p* < 0.01; ^∗∗∗^*p* < 0.001; and ^****^p < 0.0001, one-way ANOVA, Tukey’s multiple comparison).

THP1 is a human monocytic cell line used to study monocyte–macrophage differentiation, signaling, innate immune, and antiviral responses (44). It constitutively expresses the TREM2-TYROBP receptor and responds to known TREM2 stimulants, such as IL-4, similar to primary macrophages (45). Using CRISPR/Cas9 genome editing, we generated TREM2 KOs in THP1 (Supplementary Figure 3) and created doxycycline-inducible derivatives that express CV or R47H TREM2 on the KO background. To study the effect of TREM2 on the phagocytic uptake of Aβ, we treated unmodified and TREM2 KO THP1 monocytes with pHrodo-labeled, *in vitro* aggregated Aβ substrate. Consistent with previous findings in primary mouse macrophages and microglia (46), TREM2 ablation significantly reduced the capacity of human THP1 monocytes to engulf Aβ oligomers (Figure 6). Interestingly, both CV and R47H TREM2 overexpressed on the KO background were capable of restoring the Aβ phagocytic uptake.

### TREM2 Has a Stimulatory Role in the Antiviral Interferon Type I Response in Myeloid Cells

**FIGURE 7:**
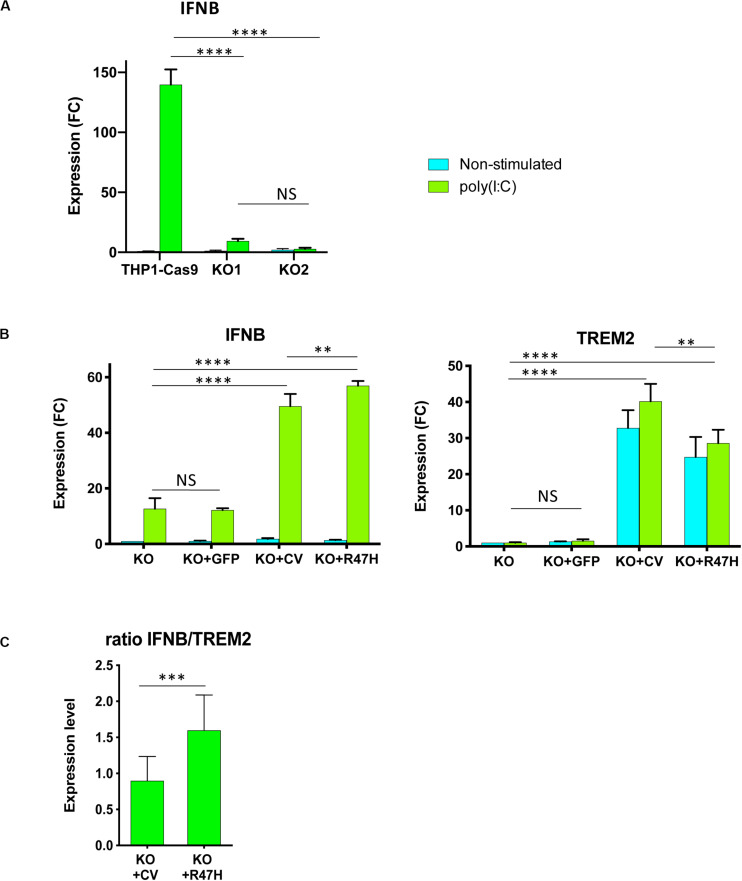
TREM2 stimulates IFN I response in THP1 cells. **(A)** Absence of TREM2 inhibited IFN I response in THP1 TREM2 KO. IFNB expression in non-stimulated cells and after 24 h with poly(I:C) complexes was measured by qRT-PCR. THP1-Cas9, THP1 stably expressing Cas9 nuclease; KO1, KO2 are two independent TREM2 KO clones. IFNB expression was normalized to the level in unstimulated THP1-Cas9. FC – fold changes. **(B)** Overexpression of TREM2, but not GFP, restored the IFN I response. Assays were performed in biological triplicates and repeated at least two times; **(A,B)** show representative experiments. KO – TREM2 KO1 cells; KO + GFP – TREM2 KO1 expressing GFP; KO + CV – TREM2 KO1 expressing common variant (CV) TREM2; KO + R47H – TREM2 KO1 expressing R47H TREM2. All cells were treated with doxycycline. TREM2 and IFNB expression was normalized to their levels in unstimulated KO. **(C)** Expression of R47H TREM2 induced a higher level of IFNB as compared with CV TREM2. Ratio of IFNB to TREM2 expression was calculated for TREM2 KO1 THP1 expressing either CV TREM2 or R47H TREM2 after 24-h poly(I:C) treatment from eight independent biological replicates performed on different days. Data are presented as mean ± SD. Significance in **(A,B)** was calculated using two-way ANOVA, Tukey’s multiple comparison test; significance in **(C)** was calculated using an unpaired two-tailed *t*-test (NS, non-significant; ^∗∗^*p* < 0.01; ^∗∗∗^*p* < 0.001; and ^****^p < 0.0001).

**FIGURE 8:**
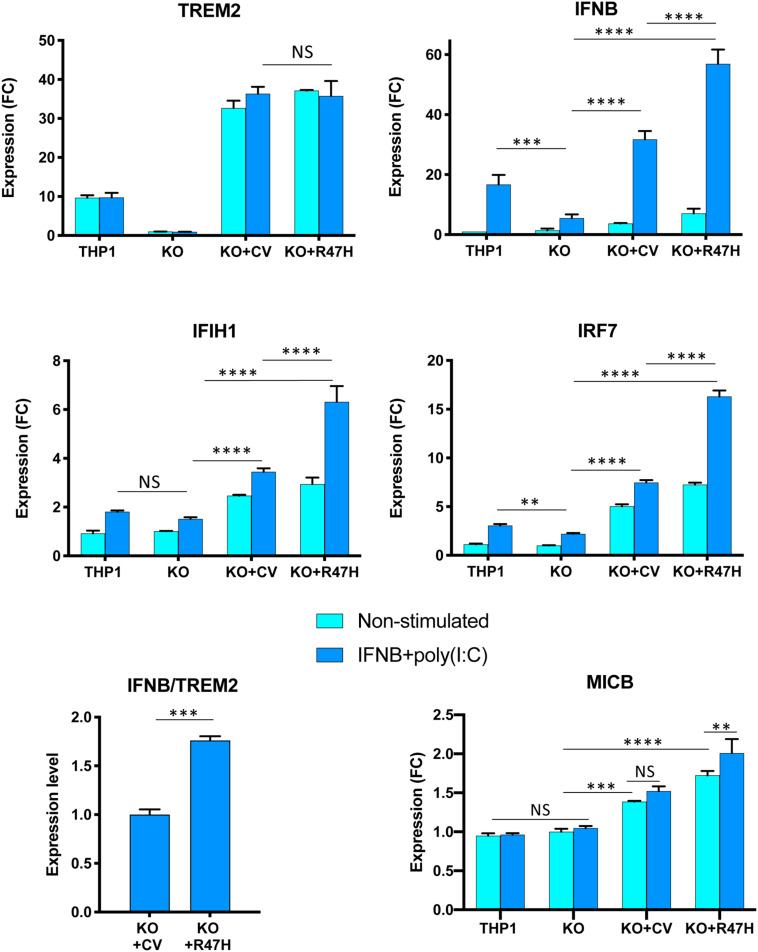
Effect of TREM2 on key interferon (IFN) response genes and the NKG2D ligand MICB upon stimulation with a combination of poly(I:C) and IFNβ. All cells were treated with doxycycline. Gene expression in non-stimulated cells and after 24 h stimulation was measured by qRT-PCR. THP1, unmodified cells; KO, TREM2 KO1 line; KO + CV, KO + R47H, TREM2 KO1 expressing common variant (CV) or R47H TREM2, respectively. Gene expression levels were normalized to expression in unstimulated KO. FC, fold changes. Assays were performed in biological triplicates and repeated two times; a representative experiment is shown. Data are shown as mean ± SD (NS, non-significant; ^∗∗^*p* < 0.01; ^∗∗∗^*p* < 0.001; and ^****^p < 0.0001, two-way ANOVA, Tukey’s multiple comparison test). Ratio of IFNB to TREM2 expression was calculated for TREM2 KO1 THP1 expressing either CV TREM2 or R47H TREM2 after 24-h poly(I:C) treatment from biological triplicates; data shown as mean ± SD (^∗∗∗^*p* < 0.001, unpaired two-tailed *t*-test).

To investigate the involvement of TREM2 in the IFN I response, THP1 derivatives were differentiated *in vitro* to macrophages with PMA (25, 26). IFN I response was induced by the high molecular weight RNA-mimetic poly(I:C) in a formulation that specifically activates the RIG-I/MDA5 (IFIH1) signaling pathway (47, 48). After 24 h of stimulation, the response was evident from the upregulation of the IFNB transcript (Figure 7A). Absence of TREM2 caused a notable decrease of IFNB stimulation in response to poly(I:C) that was restored by doxycycline-induced expression of ectopic CV or R47H TREM2 in the KO cells (Figure 7B). When normalized to the level of TREM2 expression, R47H had a higher stimulatory effect on IFNB than CV TREM2 (Figure 7C). When derivatives were co-treated with IFNβ and poly(I:C), a combination is known to potentiate the response, R47H TREM2 had a superior effect on the IFNB level, as well as on key IFN type I response genes, such as IRF7 and IFIH1 (Figure 8). Stimulation of the IFN I response did not upregulate the NKG2D ligand MICB in unmodified THP1 cells nor TREM2 KO cells, unlike upon pro-inflammatory stimulation with LPS + IFNγ (Supplementary Figure 2). Overexpression of either CV- or R47H-TREM2 in unstimulated KO cells modestly increased MICB expression, and the effect was the most pronounced in KO cells overexpressing R47H after IFN I stimulation. In aggregate, our data indicate a previously unrecognized role of TREM2 as a positive regulator of IFN I response in myeloid cells expressing this receptor.

## Discussion

Pathogenic TREM2 variants of different effects are found in several neurodegenerative diseases. In PLOSL, mutations that inactivate TREM2, including premature stop codons (49, 50), splice-site mutations (22, 51), and the missense variants D134G, K186N (4), Y38C, T66M, and V126G (5, 52, 53), have a recessive mode of inheritance. In contrast, AD-associated R47H has a dominant mode of inheritance. Whereas pathogenic missense PLOSL variants affect protein folding and stability, decreasing TREM2 surface localization, R47H, and other AD-associated mutations alter the ligand-binding interface of TREM2 (54, 55). Analysis of TREM2 R47H function in human and mouse reporter cells showed impaired recognition of certain anionic lipid ligands (20, 56). These findings are interpreted as a weakened function not compensated by the normal allele (haploinsufficiency). Assuming this scenario, heterozygous carriers of known loss-of-function TREM2 variants in PLOSL families would also have increased AD susceptibility. To date, pedigrees with nine distinct TREM2-inactivating variants have been described across the world (57), and a study of AD burden in heterozygous mutation carriers in these PLOSL families would be required to test this hypothesis. Of several homozygous R47H individuals reported in Iceland, none manifested a PLOSL-like early-onset form of dementia, indicating that the variant protein has retained essential function (2). Given all known limitations of overexpression studies, our results indicate that TREM2 R47H does not act as a loss-of-function mutant in the context of Aβ phagocytosis (Figure 6).

We observed the upregulation of immune and antiviral defense networks in the brain of heterozygous TREM2 R47H carriers who developed AD. It was exemplified by the upregulation of pro-inflammatory cytokines and activation of IFN I response, an important line of defense against viruses. This pathway is activated upon sensing viral nucleic acids inside the cell, triggering a cascade of immune responses that restrict viral propagation and eliminate infected cells. In the absence of viral infection, unchecked IFN I response is harmful, being a cause of Aicardi–Goutières syndrome (AGS), a group of monogenic interferonopathies that affect the CNS (58). A common RNA expression phenotype, an “interferon signature,” features upregulation of IFN I genes in AGS patients. There is no consensus as to the precise set of genes that constitute the signature (59), so the subsets may vary considerably between cases with different mutated genes that cause AGS and even between patients with the same mutation (60). Of note, some canonical IFN I response genes, such as STAT1, STAT2, ISG15, IFIT1, and IFIT2, were not perturbed in TREM2 R47H AD brains.

The IFN I response signature in the microglia is also a characteristic of brain aging in humans and mice (61). Pharmacologic blockade of IFN I signaling ameliorated age-related cognitive decline in mice. At present, it remains to be established which aging-associated processes may drive activation of the IFN I response in the brain. Under certain circumstances, the antiviral response may be triggered by reactivation of silenced endoretroviruses (ERVs) or other transposable elements abundant in the human genome that mimic double-strand RNA (dsRNA) produced during viral infection. For instance, DNA methyltransferase inhibition results in the demethylation of silenced ERV, and their expression triggers a suicidal IFN I response (62, 63). In aging, transposable elements undergo profound epigenetic alterations, and their reactivation is observed in the context of organismal and cellular senescence in humans and other species (64). Inducing ERV activation in the mouse brain causes significant hippocampus-related memory impairment accompanied by robust activation of antiviral immune response mediated through Irf7; the pathology is attenuated in mice lacking cytosolic viral RNA sensor protein MAVS (65).

The absence of TREM2 signaling in macrophages differentiated from KO-THP1 monocytes substantially blunted the response to viral dsRNA mimetic poly(I:C), seen as a decrease of IFNB induction. In the “add-back” experiment, the induction of recombinant TREM2 restored IFNB production. Notably, the R47H variant induced greater IFNB increase than CV protein, indicating a possible gain of function in the context of IFN I response stimulation. We propose a model in which TREM2 regulates the intensity of the antiviral IFN I response in specialized innate immune cells expressing this receptor, such as microglia and macrophages. The epigenetic reprogramming that occurs in the aging brain may activate transcription of silenced ERV or other transposable elements, e.g., through DNA demethylation or changes in histone modifications of their chromatin. Transcription from repeat elements positioned in a sense and anti-sense orientation can generate endogenous dsRNA, which is recognized as foreign and triggers chronic activation of the IFN I pathway. Trem2 is upregulated in the course of the acute response to RNA virus in mouse macrophages (15), Trem2 and TREM2 levels increase with age in mouse and human brains (66), and TREM2 is elevated in the brain of R47H carriers. The proposed stimulatory role of the TREM2 risk allele in AD pathology is consistent with the small overlap of perturbed genes we observed in PLOSL and TREM2 R47H AD brains, as well as with inhibited IFN I response in THP1 TREM2 KO.

When this manuscript was in preparation, we became aware of another study that analyzed gene expression in TREM2 R47H brains. Prokop and colleagues studied several brain areas by immunohistochemistry, followed by NanoString nCounter analysis of RNA isolated from FFPE sections (33). Notably, TREM2 R47H AD patients presented with a significant increase of senescent/dystrophic microglia in the hippocampus. R47H carriers also had elevated immune and microglial activation scores in the frontal cortex and, to a lower extent, in the hippocampus. A different choice of predesigned gene panels in this study and our work may, in part, explain the difference in the top observed immune gene signatures. The abundance of senescent microglia in TREM2 AD cases is an intriguing observation pointing to a possible role of this immune receptor in accelerated physiological aging of the microglia. In this light, our finding of co-occurrence of activated pro-inflammatory and IFN I pathways with upregulated NKG2D ligands, such as MICA/MICB and ULBP2, in TREM2 R47H AD brains may be viewed as part of complex senescence phenotype (67–69).

We acknowledge the limitations of our study engendered primarily by the rarity of autopsy samples from TREM2 R47H AD and PLOSL patients, most of which are limited to FFPE material. Notwithstanding these constraints, our findings in brains of R47H TREM2 carriers, together with demonstrated effects of TREM2 KO and overexpression in myeloid cells, encourage further testing of the hypothesis that the variant exacerbates the neurodegenerative process in AD via chronic activation of the antiviral immune defense.

## Data Availability Statement

The raw data supporting the conclusions of this article will be made available by the authors, without undue reservation, to any qualified researcher.

## Ethics Statement

The studies involving human participants were reviewed and approved by Institutional Review Board of the University of Washington. The patients/participants provided their written informed consent to participate in this study.

## Author Contributions

OK, KK, and WR: conceptualization. AR, KK, MD, and TKB: methodology. OK, KK, AR, MM, NB, and W-MC: investigation. J-IS, CK, and TKB: resources. OK and KK: writing—original draft. TDB and WR: writing—review and editing. OK, AR, and KK: visualization. OK and WR: supervision. All authors contributed to the article and approved the submitted version.

## Conflict of Interest

The authors declare that the research was conducted in the absence of any commercial or financial relationships that could be construed as a potential conflict of interest.

## Footnotes

1 http://metascape.org

2 http:/string-db.org
