# Supplementary material for: Triggering Receptor Expressed on Myeloid Cell 2 R47H Exacerbates Immune Response in Alzheimer’s Disease Brain
Source: Front Immunol. 2020 Sep 25;11:559342. doi: 10.3389/fimmu.2020.559342 (PMC7546799; doi:10.3389/fimmu.2020.559342)
Supplement: Supplementary file 1 [file Data_Sheet_1.PDF]

Table S1. Clinical and neuropathological characteristics of cases and controls.

Subject ID	Description	Gender	Age at death	Affected status	Braak	CERAD	Contributing pathology / affected region	Gene mutation	Transcription profiling	Reference
458	Non-affected R47H TREM2	F	87	Non AD	III (B2)	C0		TREM2 R47H	RNA-seq	(Korvatska et al., 2015)
414	TREM2 R47H AD	F	76	AD	VI (B3)	C1		TREM2 R47H	RNA-seq, Nanostring	
5799	TREM2 R47H AD	F	64	AD				TREM2 R47H	Nanostring	
661	TREM2 R47H AD	M	60	AD	VI (B3)	C3	LB, neocortical (diffuse)	TREM2 R47H	Nanostring	
5790	TREM2 R47H AD	F	85	AD	VI (B3)	C2		TREM2 R47H	Nanostring	(Korvatska et al., 2015)
1205	TREM2 R47H AD	F	66	AD	V (B3)	C3		TREM2 R47H	Nanostring	(Korvatska et al., 2015)
1462	TREM2 R47H AD	F	89	AD	V (B3)	C3	LB, amygdala predominant	TREM2 R47H	Nanostring	(Korvatska et al., 2015)
1558	TREM2 R47H AD	M	67	FTLD, AD		C2	Tauopathy	TREM2 R47H	Nanostring	
4731	TREM2 R47H AD	F	81					TREM2 R47H	Nanostring	
520	sAD	F	67	AD	VI (B3)	C3			Nanostring	
1291	sAD	M	67	AD	VI (B3)	C3			Nanostring	
655	sAD	M	63	AD	VI (B3)	C3	LB, amygdala predominant		Nanostring	
297	sAD	F	62	AD	VI (B3)	C3			Nanostring	
5918	sAD	F	81	AD	VI (B3)	C3			Nanostring	
5964	sAD	F	85	AD	VI (B3)	C2			Nanostring	
1135	sAD	F	76	AD	V (B3)	C3			Nanostring	
461	sAD	F	96	AD	VI (B3)	C3	LB, amygdala predominant		Nanostring	
5805	sAD	F	64	AD	VI (B3)	C3			Nanostring	
220	sAD	F	83	AD	V (B3)	C3			Nanostring	
226	sAD	F	77	AD	V (B3)	C3	LB, brainstem predominant		Nanostring	
5986	sAD	F	64	AD	VI (B3)	C2			Nanostring	
1222	sAD	M	65	AD	VI (B3)	C2	LB, neocortical (diffuse)		Nanostring	
600	sAD	F	91	AD	VI (B3)	C3			Nanostring	
3087	sAD	M	68	AD	VI (B3)	C3			Nanostring	
NHD 1	PLOSL	M	50	PLOSL	NA	NA		TREM2 D134G	Nanostring	(Paloneva et al., 2002)
NHD 2	PLOSL	F	48	PLOSL	NA	NA		TYROBP c.141delG	Nanostring	(Satoh et al., 2014)
NHD 5	PLOSL	M	38	PLOSL	NA	NA		TYROBP c.141delG	Nanostring	(Satoh et al., 2014)
NHD 7	PLOSL	M	39	PLOSL	NA	NA		TREM2 c.482+2T>C	Nanostring	(Numasawa et al., 2011)
752	CNT	M	87	CNT	0 (B0)	C0			RNA-seq	
2060	CNT	F	91	CNT	II (B1)	C1			RNA-seq	
750	CNT	F	93	CNT	I (B1)	C0			RNA-seq	

431	CNT	M	86	CNT	II (B1)	C0			Nanostring	
1845	CNT	F	73	CNT	II (B1)	C0			Nanostring	
420	CNT	F	87	CNT	II (B1)	C0			Nanostring	
1308	CNT	F	80	CNT	III (B2)	C2			Nanostring	
6049	CNT	M	56	CNT					Nanostring	
1988	CNT	F	76	CNT	II (B1)	C0			Nanostring	
591	CNT	F	89	CNT	III (B2)	C1			Nanostring	
1362	CNT	M	72	CNT	0 (B0)	C0			Nanostring	
310	CNT	M	87	CNT	I (B1)	C1			Nanostring	
1854	CNT	F	77	CNT	III (B2)	C1			Nanostring	
2040	CNT	F	77	CNT	III (B2)	C0			Nanostring	
1321	CNT	F	77	CNT	I (B1)	C1			Nanostring	
1389	CNT	M	71	CNT	II (B1)	C0			Nanostring	
2044	CNT	F	94	CNT	II (B1)	C0			Nanostring	
750	CNT	F	93	CNT	I (B1)	C0			Nanostring	
2599	CNT	F	71	CNT	I (B1)	C0			Nanostring	
224	CNT	F	70	CNT	0 (B0)	C0			Nanostring	

Abbreviations:
M - male
F - female
LB - Lewy Bodies
PD - Parkinson's disease
FTLD - Frontotemporal lobar degeneration
NA - not applicable
