# Supplementary material for: Triggering Receptor Expressed on Myeloid Cell 2 R47H Exacerbates Immune Response in Alzheimer’s Disease Brain
Source: Front Immunol. 2020 Sep 25;11:559342. doi: 10.3389/fimmu.2020.559342 (PMC7546799; doi:10.3389/fimmu.2020.559342)
Supplement: Supplementary file 2 [file Data_Sheet_2.PDF]

Table S2. Neuropathologic, demographic and RNA QC characteristics of the groups for Nanostring gene expression analysis.

	CNT (N=17)	sAD (N=15)	TREM2 R47H AD (N=8)	PLOSL (N=4)
BraaK: AVE (min-max)	1.8 (0-3)	5.7 (5-6)	5.5 (5-6)	NA
CERAD: AVE (min-max)	0.4 (0-2)	2.8 (2-3)	2.3 (1-3)	NA
AGE, years: Ave (min-max)	79 (56-94)	74 (62-96)	74 (64-89)	42 (38-48)
Gender: males, %	29,4%	27%	25%	75%
PMI, hours: AVE (min-max)	10 (2.5-22.2)	12 (2.5-36)	13 (7.6-18)	ND
RIN: AVE (min-max)	2.5 (1.6 - 4.5)	2.4 (2.1-3.1)	2.4 (2-2.8)	2.4 (2.2-2.6)

NA – not applicable
ND – not determined
