# Supplementary material for: Triggering Receptor Expressed on Myeloid Cell 2 R47H Exacerbates Immune Response in Alzheimer’s Disease Brain
Source: Front Immunol. 2020 Sep 25;11:559342. doi: 10.3389/fimmu.2020.559342 (PMC7546799; doi:10.3389/fimmu.2020.559342)
Supplement: Supplementary file 3 [file Data_Sheet_3.PDF]

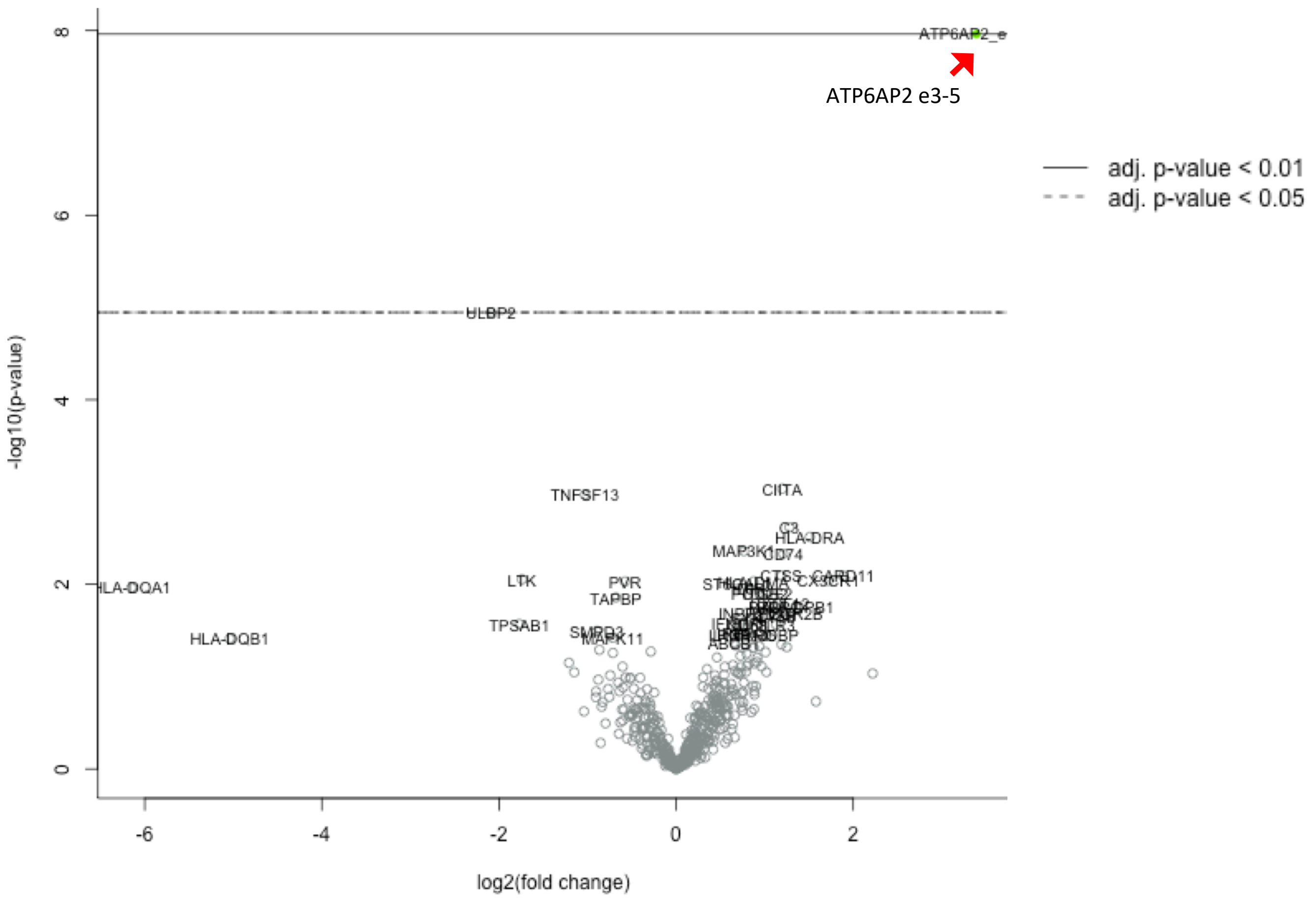

Figure S1. Nanostring nCounter detects upregulated abnormal ATP6AP2 e3-e5 splice isoform in RNA samples isolated from hippocampal FFPE sections of the ATP6AP2 mutation carriers.

Volcano plot displaying DE genes in carriers (N=2) vs a baseline of gene expression in controls (N=17).

Red arrow depicts position of ATP6AP2_e3–e5 splice-isoform.

The y-axis corresponds to the $\log_{10}$(p-value), and the x-axis displays the $\log_{2}$(fold change) value.

Carriers: female, 90 years; PMI: 25h, RIN :4.6; male, 86 years; PMI: ND; RIN: 1.8

Control subjects are listed in Tables S1, S2. A probe to e3-e5 junction of the *ATP6AP2* gene was designed as a part of PanelPlus Code Set and analyzed with a pre-designed Pan-Cancer Immune Panel.

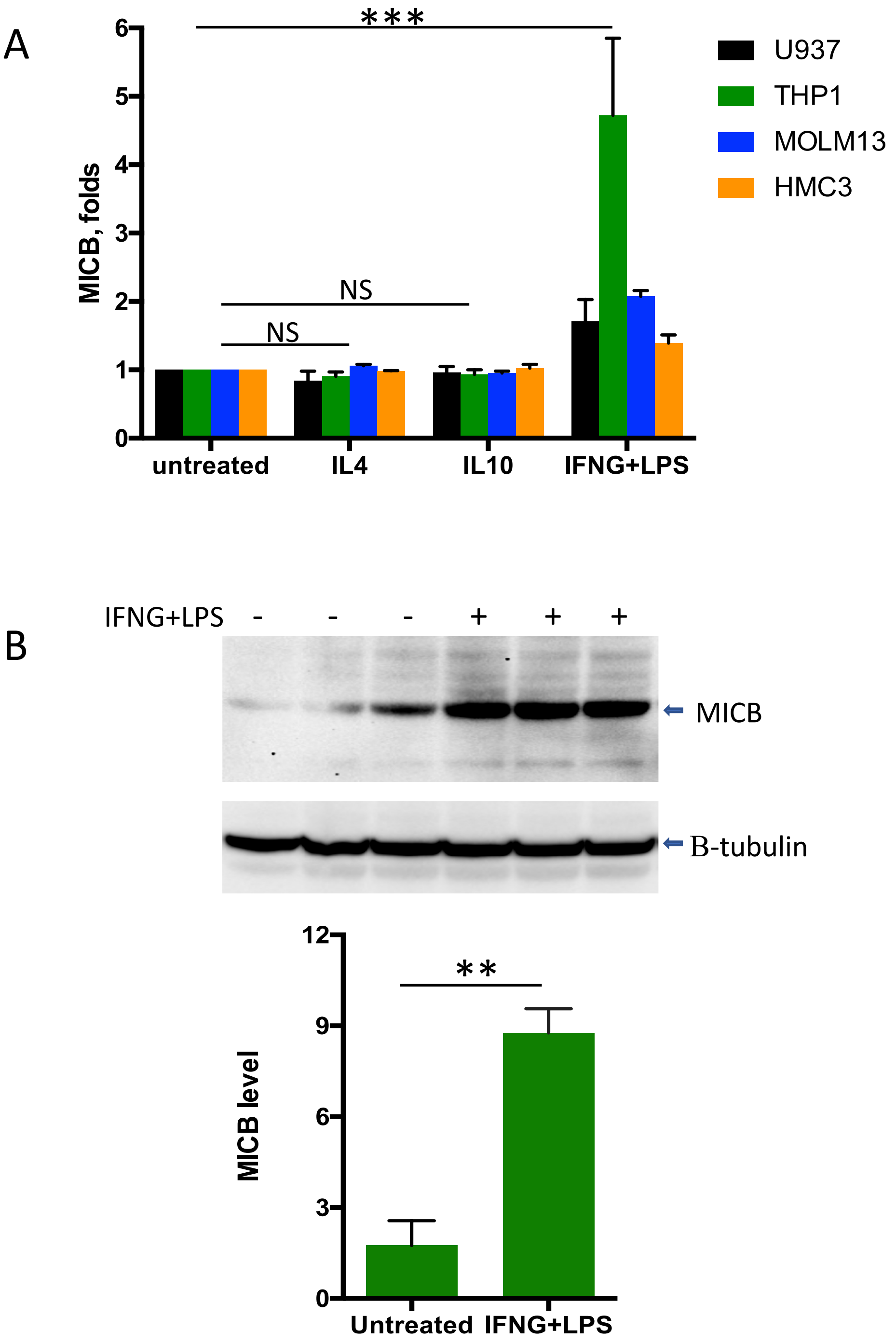

Figure S2. Pro-inflammatory stimulation leads to up-regulation of stress-ligand MICB in microglial (HMC3) and blood (THP1, MOLM13, U937) myeloid cell lines.
(A) MICB mRNA expression was measured by qRT-PCR in cells treated with of IL4, IL10 or a combination of LPS with IFN–γ for 24h. MICB expression was normalized to its level in untreated cells. Data presented as mean ± SEM (*** - p-value < 0.001, two-way ANOVA, Dunnett's multiple comparison test)
(B) MICB protein expression in THP1 untreated or treated with LPS and IFN–γ for 24h was measured by Western blotting and normalized to Beta-tubulin. Data presented as mean ± SEM (** - p-value < 0.01, paired two-tailed T-test)

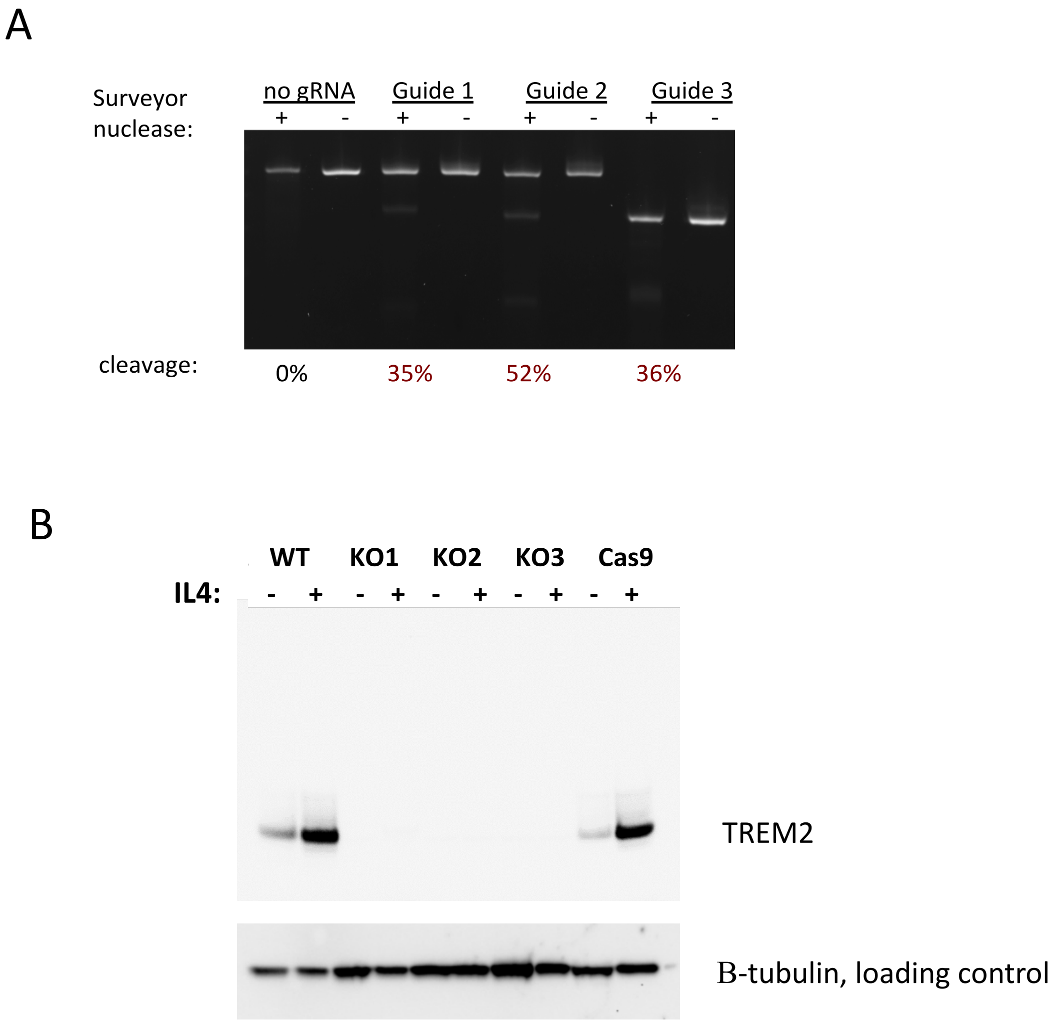

Figure S3. CRISPR/Cas9-mediated genome editing in THP-1 cells.
(A) Efficiency of cleavage by different guide RNAs evaluated by Surveyor nuclease assay.
(B) Expression of TREM2 protein in THP-1 (WT), cells stably expressing Cas9 nuclease and three independent knockout clones of TREM2 (KO1-3). TREM2 level in cell extracts was measured by Western blot. TREM2 expression was stimulated with IL4.
